# The Role of Imaging Biomarkers in the Assessment of Sarcopenia

**DOI:** 10.3390/diagnostics10080534

**Published:** 2020-07-30

**Authors:** Roberto Sanz-Requena, Francisco Miguel Martínez-Arnau, Ana Pablos-Monzó, Cristina Flor-Rufino, Joaquín Barrachina-Igual, Gracián García-Martí, Luis Martí-Bonmatí, Pilar Pérez-Ros

**Affiliations:** 1 Radiology Department, Hospital Quironsalud Valencia, 46010 Valencia, Spain; roberto.sanz@quironsalud.es (R.S.-R.); gracian.garcia@quironsalud.es (G.G.-M.); luis.marti@quironsalud.es (L.M.-B.); 2 Department of Physiotherapy, Universitat de Valencia, 46010 Valencia, Spain; cristina.flor@uv.es; 3 Frailty and Cognitive Impairment Research Group (FROG), University of Valencia, 46010 Valencia, Spain; 4 Faculty of Physical Activity and Sport Sciences, Universidad Católica de Valencia San Vicente Mártir, 46900 Valencia, Spain; ana.pablos@ucv.es; 5 Doctoral School, Universidad Católica de Valencia San Vicente Mártir, 46002 Valencia, Spain; joaquin.barrachina@ucv.es; 6 Nursing Department, Universidad Católica de Valencia San Vicente Mártir, 46007 Valencia, Spain; pilar.perez@ucv.es

**Keywords:** sarcopenia, magnetic resonance imaging, biomarker, older people

## Abstract

Background: The diagnosis of sarcopenia through clinical assessment has some limitations. The literature advises studies that include objective markers along with clinical assessment in order to improve the sensitivity and specificity of current diagnostic criteria. The decrease of muscle quality precedes the loss of quantity, so we studied the role magnetic resonance imaging biomarkers as indicators of the quantity and quality of muscle in sarcopenia patients. Methods: a cross-sectional analysis was performed to analyze what MR-derived imaging parameters correlate better with sarcopenia diagnostic criteria in women of 70 years of age and over (independent walking and community-dwelling women who were sarcopenic in accordance with EWGSOP criteria with muscle mass adjusted to Spanish population were chosen). Results: The study included 26 women; 81 ± 8 years old. A strong correlation was obtained between cineanthropometric variables (BMI; thigh perimeter and fat mass) and imaging biomarkers (muscle/fat ratio, fatty infiltration, muscle T2*, water diffusion coefficient, and proton density fat fraction) with coefficients around 0.7 (absolute value). Conclusions: Knowing the correlation of clinical parameters and imaging-derived muscle quality indicators can help to identify older women at risk of developing sarcopenia at an early stage. This may allow taking preventive actions to decrease disability, morbidity, and mortality in sarcopenia patients.

## 1. Introduction

Sarcopenia is defined by the European Working Group on Sarcopenia in Older People (EWGSOP) as a disorder described by the progressive loss of skeletal muscle mass and strength, with an increased risk of adverse events such as physical disability, loss of quality of life, and death [1]. Primary sarcopenia is the loss of muscle mass and function in a chronic and progressive manner related to the aging process, whereas secondary sarcopenia is associated with causal factors (nutritional, activity, derived from some pathology or aging). According to the EWGSOP, sarcopenia can be assessed by means of indirect measures of muscle function and muscle mass [1]. For the diagnosis of sarcopenia, at least two criteria must be met: low lean mass and a functional loss of either palmar grip strength or mobility [1,2]. The assessment of functional mobility is related to the speed of walking, measured through timed up and go (TUG) or the short physical performance battery (SPPB), where speeds lower than 1 m/s are related to the increase of disability, hospital admissions, falls, institutionalization, and early death [2].

The loss of muscle function that accompanies aging is inevitable. The muscular mass is lost in a linear and progressive way and can decrease by 25% when reaching 75–80 years. Aging is associated not only with changes in muscle mass, but also with muscle composition, contractility, the properties of the constituent components of muscle and tendons. Between 50–60 years, strength decreases annually by 1.5%, reaching up to 3% from the age of 60. These changes can be due to the decrease of contractile elements, the loss of motor units, the reduction of the total number of muscle fibers, and the reduction of type II fibers by transformation into type I fibers, with the consequent loss of muscle power [3]. Therefore, the decrease in strength is the main condition that causes the dependence of older people, regardless of other variables. This hampers the most diverse tasks of everyday life, in addition to contributing to the loss of balance that implies a propensity to fall, causing bone fractures and disability in many cases [4].

The diagnosis of sarcopenia through clinical assessment has its limitations. Bio-electrical impedance analysis (BIA) can be influenced by factors such as age, sex, hydration status, and ethnicity [5]. Functional tests and grip strength can be affected by comorbidity, joint problems, and neurological deficits [6]. Age-related aspects such as the loss of vertebral disc thickness and the consequent decrease in height can affect body mass index (BMI). A recent study compared the diagnosis of sarcopenia based on the EWGSOP criteria published in 2010 with the recent EWGSOP2 published in 2019, finding differences, with the EWGSOP2 criteria showing moderate sensitivity (41.9%) and high specificity (79.3%) in relation to the first [7]. This, coupled with the existence of several definitions of sarcopenia worldwide, with three major working groups considering different criteria [7] results in disparate rates for the prevalence of sarcopenia, ranging between 9.9% and 40.4%. Besides, these figures differ according to sex, despite the fact that both the rates for muscle mass and strength for sarcopenia are adjusted to normative values by sex [8].

This diagnostic variability is due to the lack of objective indicators that accurately relate the pathophysiological basis of sarcopenia to the clinical condition of the patients. For this reason, the literature advises studies that include objective markers along with clinical assessment in order to improve the sensitivity and specificity of current diagnostic criteria. The most objective markers for diagnosis are imagining markers [9,10].

Magnetic resonance imaging (MRI) is considered the reference for the non-invasive assessment of the muscle. It allows differentiating the muscle and the fat with very high contrast and it can be used to quantify the muscle volume, the amount of intramuscular fat infiltration and other biochemical indicators related to muscle quality [11]. As it uses non-ionizing radiation, it is suitable as a follow-up technique in patients under treatment. Despite its higher cost, there is currently a broad consensus on the role of quantitative MRI as a key element to validate the results in musculoskeletal clinical trials [12].

From a quantitative point of view, imaging biomarkers are increasingly relevant for the diagnosis and longitudinal evaluation of treatments [13]. In sarcopenia, it is convenient to assess both the quantity and quality of the muscle, as quality reduction precedes muscular volume loss [3]. Quality assessment includes the calculation of imaging biomarkers that assess tissue hydration (transverse relaxation time (T2) and water apparent diffusion coefficient (ADC), which increase in association with inflammatory processes and edema [14]), microscopic fat fraction (using MRI multiecho specific techniques [15]) and muscle homogeneity with texture analysis [16]. To date, few studies incorporate these MRI biomarkers as objective criteria to study sarcopenia. As far as we know, no study has provided a comprehensive analysis combining traditional clinical/biometrical parameters and imaging biomarkers. A recent review concludes with the need for studies including both MRI and clinical parameters for the standardization of diagnostic criteria and the early detection of sarcopenia, especially in older populations [17]. 

We hypothesize that the inclusion of MRI biomarkers in the management of sarcopenia patients will provide relevant insights for the diagnosis of this disorder. Our aim is to analyze what quantitative and qualitative MRI parameters correlate better with EWGSOP criteria in order to complement the diagnosis of sarcopenia and set a baseline for the assessment of longitudinal therapeutic interventions.

## 2. Materials and Methods 

### 2.1. Study Design

This work was a cross-sectional analysis of the baseline data of the SarcoImage study (ClinicalTrials.gov reference NCT03834558). Between January and February 2019, we conducted cineanthropometric, functional, and MRI assessments targeting a sample of community-dwelling older women with sarcopenia located at the city of Valencia, Spain. 

### 2.2. Patient Selection

Eligible patients complied with the following criteria: women of 70 years or over; independently walking (may have technical aids, but not from another person); community-dwelling with habitual residence in the Hospital Clínico Universitario de Valencia area; subjects who were sarcopenic in accordance with EGWSOP criteria with muscle mass adjusted to Spanish population [18].

Patients were excluded if they had severe visual or auditory deficits, severe psychiatric illness (severe depression subjected to treatment, or acute psychosis), and moderate to severe cognitive impairment (diagnosed previously by a physician) or contraindications for the MRI study, especially carriers of non-compatible pacemakers, neurostimulators, cochlear implants, and intracranial aneurysm clamping.

After initial screening in primary care centers close to the intervention center by their referring physicians, 110 people were assessed. From this eligible group, 84 subjects were excluded: 62 did not meet the criteria for sarcopenia, 4 lived far from the intervention center, 4 were acutely ill at the time of assessment, 2 were cognitively impaired, 3 could not undergo the MRI exploration, and 9 did not agree to sign the informed consent.

All the subjects included in the study signed an informed consent to participate and the Local Ethics Committee approved the study protocol (H1488746567568—Ethics Committee of University of Valencia—6th April 2017).

### 2.3. Clinical and Functional Parameters

For each patient, a comprehensive geriatric assessment was conducted by a nurse, two physiotherapists and two sport sciences professionals. The following variables were collected:

Demographic variables: age and gender.

Sarcopenia: muscle mass, handgrip strength, and functional mobility (EWGSOP criteria 2010) [2].

Cineanthropometric: Height was measured to the nearest 0.1 cm using a Seca 200 scale with an attached stadiometer (Seca, Hamburg, Germany). Calf and thigh perimeters were measured with a metric tape with a millimeter scale (Lufkin w606 PM; Cooper Industries, Lexington, Surrey, Canada). The protocols were those established by the International Society for Advancement of Kinanthropometry [19]. Body weight (±0.1 kg) was measured using a BC-418 MA^®^ BIA device (Tanita Corp, Tokyo, Japan) [20]. The BIA device provided measurements of impedance (±1 Ω) and estimates of BMI (±0.1 kg·m^−2^), muscle mass (±0.1 kg) and fat mass (±0.1 kg), and skeletal muscle mass index (±0.1 kg·m^−2^) calculated as muscle mass/height^2^. BIA measurements were carried out in the early morning following the protocol indicated by Martínez [21], and at a frequency of 50 kHz and 550 mA. Dominant hand dynamometry was measured with a JAMAR dynamometer (Lafayette Instrument Company, Lafayette, IN, USA) using the protocol described in previous research [22].

Functional: to classify the participants based on the physical activity performed (low vs. moderate) the International Physical Activity Questionnaire (IPAQ) scale (with Cronbach’s alpha 0.914) [23] was used. Functional performance in activities of daily living was registered by Barthel scale (with Cronbach’s alpha 0.70) [24]. To assess the nutritional status, the mini nutritional assessment scale was used (MNA-SF with Cronbach’s alpha 0.670) [25]). The SPPB scale (with Cronbach’s alpha 0.76) was used to assess the physical performance of the lower extremities, including of three tests: a balance test, walking test and repeated chair stand test [26]. Maximum dominant leg strength was measured through 3 exercises: maximum isotonic knee extension [27], maximum isotonic leg press [28], and maximum isometric knee extension [24]. Maximal isotonic contractions were measured using F&H Fitness Gym machines (F&H Fitness Gym Equipments, Castellón, Spain). Repetitions to fatigue for each exercise were determined by assigning each participant a percentage of her 1-RM, ranging from 75% to 90%, and then applying Brzycki prediction equation [29]. Maximal isometric contractions were assessed using a hand-held dynamometer (model 01165, LaFayette, LA, USA). Respiratory function was assessed by performing forced spirometry (In2itive Vitalograph, Lenexa, KS, USA) to obtain spirometric parameters in accordance with international standards [30]. Finally, maximum inspiratory and maximum expiratory pressures generated by respiratory muscles in the mouth were assessed using a digital respiratory dynamometer (MicroRPM, CareFusion, Basignstoke, UK), also following the international standards [31].

MRI acquisition and analysis: MRI studies were performed within less than one week from the clinical and functional assessments. A 3 Tesla unit was used (Philips Achieva, Philips Healthcare, Best, The Netherlands), in order to obtain the best image quality and the maximum possible spatial resolution. Although whole-body MRI studies can be performed, the need to use advanced specific sequences makes it necessary for the study to focus on a particular area of the body. Therefore, the study focused on the thigh area, covering from the femoral head to the femoral condyles, so that a complete and detailed volumetric study of the muscle of both thighs could be carried out and used as baseline for longitudinal studies. A flexible 16-channel phase-array coil was used to homogenize the signal throughout the field of vision and ensure complete anatomical coverage.

**Figure 1:**
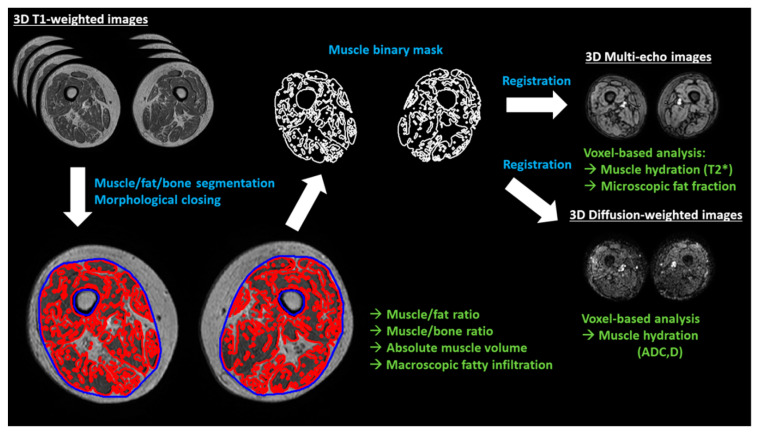
Summary of the image analysis workflow. Muscle, fat, and bone are segmented from the T1-weighted images using differences in image intensities. The muscle binary mask is then closed to calculate the macroscopic fatty infiltration (relative volume of fat enclosed in the muscle volume, excluding bone volume). Binary volumetric masks of the muscle are spatially registered to the rest of MR images (multi-echo and diffusion-weighted), obtaining microscopic fat infiltration values (fat fraction) and hydration measurements (T2*, ADC (apparent diffusion coefficient) and D (diffusion)) on a voxel basis. White text indicates MRI sequences, blue indicates image analysis processes and green is for imaging biomarkers.

The acquired MRI sequences and the corresponding imaging biomarkers are described in detail in Table 1. MR images were segmented by means of intensity clustering and tissue connectivity, so that fat, muscle, cortical bone, and marrow bone were separated. Other tissues such as blood vessels or the neurovascular bundle were excluded from the analysis, even though they represent a small part of the total volume. A summary of the image analysis workflow is presented in Figure 1.

All image analysis tasks were performed using in-house developed software (Matlab R2017a, The Mathworks Inc., Natick, MA, USA).

### 2.4. Statistical Analysis

Reference values were obtained for each parameter in order to establish the baseline metrics for the longitudinal studies. For those parameters obtained on a voxel-by-voxel approach, the mean was calculated for each patient.

The relationship between the clinical/functional parameters and the imaging biomarkers was studied with Pearson’s (normal distributions) or Spearman’s (non-normal distributions) correlation coefficients for continuous variables and with Student’s *t*-(normal) Mann–Whitney’s U-(non-normal) tests for categorical vs. continuous parameters.

The statistical analysis was performed with Matlab (R2017a, The Mathworks Inc., Natick, MA, USA) and SPSS (v19, IBM, Coppell, TX, USA).

## 3. Results

Twenty-six patients were included in the study (all female, 81 ± 8 years old). The results for each parameter are presented in Table 2. The analyzed sample presented high functionality, overweight according to BMI category and normal respiratory function.

**Figure 2:**
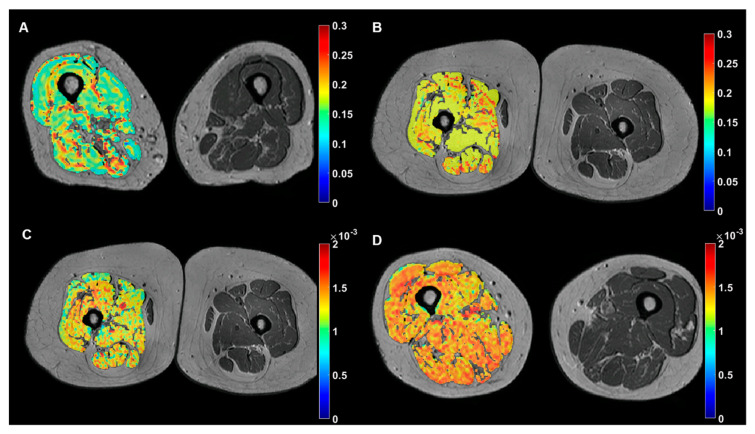
Examples of representative color-coded imaging biomarkers of the dominant leg muscle overlapped on anatomic MR images of the thighs. Top row: microscopic fat infiltration (PDFF, no units) maps showing lower values for a subject with relatively low fat mass (**A**) in comparison with a subject with high fat mass (**B**). Bottom row: muscle hydration maps ((**D**), mm^2^/s) showing lower values for a subject with IPAQ 1 (**C**) in comparison with a subject with IPAQ 2 (**D**).

There was a strong correlation between cineanthropometric variables (BMI, thigh perimeter, and fat mass) and macroscopic fatty infiltration, muscle/fat ratio, muscle/bone ratio, muscle hydration (ADC, D and T2*), and microscopic fatty infiltration (PDFF), with coefficients around 0.7 (absolute value) (Table 3, Figure 2).

Correlations between functional variables (physical strength and respiratory performance) and imaging metrics were lower, with most values ranging from 0.5 to 0.6 (absolute values) (Table 4 and Table 5)

The Mann–Whitney’s U analysis released significant differences between functional IPAQ categories and muscle hydration D, with lower hydration values associated to low physical activity subjects, D values of 0.81 ± 0.08 and 0.90 ± 0.15 •10^−3^ mm^2^/s (*p* = 0.014), for low (*n* = 8) and moderate (*n* = 18) IPAQ, respectively (Figure 2).

## 4. Discussion

Sarcopenia is currently diagnosed with functional and clinical biomarkers. The role of other biomarkers, such as MR-derived imaging biomarkers, has received less attention because MRI is a less accessible technique. This study has proposed a comprehensive approach including state-of-the-art image acquisition and analysis techniques to assess both the quantity and quality of the thigh muscles. MRI is the gold standard for the assessment of the musculoskeletal system since it provides excellent spatial and contrast resolutions for the assessment of both morphological and biochemical properties of the muscles. This allows obtaining non-invasive accurate insights of the intramuscular fat and water contents, which cannot be reached by BIA or dual X-ray absorptiometry (DXA). However, due to its high cost and difficulty of access and analysis, cineanthropometric parameters are used in daily clinical practice to diagnose sarcopenia.

The diagnosis of sarcopenia through clinical assessment has some limitations due to physical modifications related to the aging process. The current diagnostic criteria for sarcopenia are based on aspects of muscle quantity and function, but in sarcopenia, both muscle quantity and quality must be measured because loss of muscle volume is preceded by loss of muscle quality [14]. Considering qualitative muscle assessment (hydration measurements (ADC, D, and T2*), muscle microscopic fat deposits (PDFF), and fatty infiltration), we observed moderate to strong correlations with cineanthropometric, functional and respiratory parameters.

MRI-based muscle hydration measurements (ADC, D, and T2*) showed a negative correlation with the bioimpedance-derived fat mass, muscle mass, thigh perimeter, and BMI, demonstrating that muscle properties change with the total amount of fat, showing decreased values of hydration (ADC, D, and T2*) in subjects with more fat mass and vice versa. In skeletal muscles, hydration indicators (T2* and ADC/D) are related to the amount of extracellular and intracellular space, so they increase with edema [34] and inflammatory processes [35]. Additionally, hydrated volume in the lower leg decreases with age significantly [36]. In particular, T2* values are also influenced by changes in fiber architecture related to muscle aging [37] and to the presence of adipose tissue [38]. We hypothesize that subjects with less fat mass showed higher values of hydration (increased T2* and ADC) because the muscle metabolic activity is more preserved [15] and muscle quality is higher. However, longitudinal assessments are necessary to validate these results.

Muscle microscopic fat deposits (PDFF) have a positive correlation with the bioimpedance-derived fat mass, muscle mass, thigh perimeter, and BMI. Other studies have demonstrated the accuracy of microscopic fat measurements to estimate absolute fat mass [39] or an increased sensitivity to assess muscular dystrophy progression over standard functional evaluation [13,40]. In a comparison between young healthy subjects and patients with Duchenne muscular dystrophy, microscopic fat was elevated in the second group, presenting a good correlation with total fat mass and BMI [41]. These patients also showed areas of edema with increased muscle hydration, measured with T2*. Our results of thigh microscopic fat are in the range of those presented by Grimm et al. [42] for an older cohort of sarcopenia patients. These results show that this is an excellent biomarker of muscle quality, suitable as an endpoint for clinical trials. In comparison to a voxel-based microscopic assessment, both bioimpedance methods and visual scoring of T1-weighted images [43,44] are more limited, since they can only detect significant macroscopic changes or are prone to inter- and intra-observer variability, hindering the early assessment of treatments.

Considering quantitative muscle assessment (muscle volume, muscle-fat ratio, and muscle-bone ratio), we obtained significant correlations between bioimpedance-derived fat mass and MR-derived muscle/fat ratio (negative) and muscle/bone ratio (negative); and between thigh perimeters and muscle/fat ratio (negative). These results show that the fat-related bioimpedance parameters and MR-derived imaging biomarkers are in good agreement. However, the relationship between muscle-related bioimpedance parameters and MR-derived imaging biomarkers is not as straight, with a relatively weak correlation between bioimpedance-derived muscle mass and muscle/fat ratio, and no relevant correlations with muscle/bone ratio or total muscle volume. These differences may be due to the fact that bioimpedance accuracy strongly relies on the type of equations used for skeletal mass estimation [45], the state of hydration at the time of the test which may act as a confounding factor [46], or that the bioimpedance measurements correspond to the entire body while the MR analysis is locally focused on the thigh.

Our results showed increased hydration values for the group of subjects with moderate physical activity (IPAQ) in comparison to those with low IPAQ score. This demonstrates that those subjects with better muscle quality show higher hydration values measured with MRI-derived D biomarkers. No further relationships could be established for other quality-related imaging biomarkers, so these results still need validation in future studies.

In comparison with the cineanthropometric parameters, we obtained weaker correlations between imaging biomarkers and the functional parameters related to strength and respiratory performance. The total muscle volume showed a positive correlation with handgrip and SPPB. Muscle/bone ratio presented a similar trend with SPPB, reinforcing a direct relationship between the amount of muscle and physical performance. However, both muscle/fat and muscle/bone ratios showed a negative trend when compared with maximum and mean isometric knee extension contraction. This leads us to believe that the role of fat on muscle function is not clear. Some results support that fat mass could not negatively influence muscle strength, although it may negatively affect physical performance or function [47]. Other authors affirm that the presence of intramuscular fat can interfere with the maximal activation of muscle to produce an effort, while at the same time it could be a hindrance to the improvement of the quality of the muscle when resistance training is applied [48]. This suggests that muscle activation is more related to fat rather than to the lean cross-section area (CSA). The lack of a relationship between central activation and lean tissue reinforces the notion that lean tissue alone does not strongly predict neuromuscular function [49]. Further research is needed to understand the role of intramuscular fat in muscle performance.

The analysis of the respiratory parameters showed a normal respiratory function in the study population. When analyzing the relationship with imaging biomarkers, negative correlations were found for several spirometric parameters. These results cannot be conclusive given the high multifactorial modulation that these components usually present in older people. However, there are three parameters that allow us to analyze in a more analytical way the behavior of the respiratory muscles, since they are considered as determinants of respiratory sarcopenia: peak expiratory flow (PEF) [50,51] and maximum respiratory pressures (MIP and MEP) [52]. We found positive correlations between muscle volume and PEF and MEP. These parameters are both effort-dependent of the expiratory and the inspiratory musculature, as they show the relationship between the performance of lung inflation and the expiratory effort [53,54]. The presence of infiltrated fat, and its impact on muscle hydration, results in negative correlations with PEF, MIP, and MEP, and can confirm the relationship of respiratory strength parameters both with quantitative and qualitative image variables.

Our original approach to quantify macroscopic fatty infiltration of both thighs used 3D automatic techniques to enclose the muscle area and assess the fat proportion within. It allowed a fast and reproducible segmentation of the fat, the muscle, and the bone. This is an improvement over manually segmented representative CSA, as segmenting full volumes is time-consuming without automatic tools [13,55]. The performance of CSA to predict muscle has shown disparate results, with some studies releasing a volume error of less than 10% [56] and others showing unacceptable results [57]. It seems that the accuracy of CSA as a predictor of whole muscle volume may depend on the body part under study [58].

All the methods for skeletal muscle assessment have advantages and disadvantages, but the use of MRI introduces a gold standard that allows a better understanding of anthropometric methods. MRI data increase the knowledge of muscle quality, providing detailed information about the amount and composition of both individual and compartmentalized muscles [59]. Muscle mass and functionality do not always match during the aging process. In some cases, other body compartments such as fat mass are better predictors of outcome in older subjects than skeletal muscle mass [60]. As muscle in people with sarcopenia is characterized by an increased fat component, particularly in the lower extremities [61,62], this component should be taken into account when assessing sarcopenia.

Our study has several limitations. MR whole-body imaging with both morphological and functional imaging acquisition sequences is time-consuming. Therefore, our decision was to focus on the thighs as a representative area of the subjects’ physiological status. The different muscle compartments of the thigh might have different behaviors, requiring accurate sub-segmentation of the thigh muscles [63,64]. However, this is still a difficult task to automate, especially in patients with sarcopenia, where muscles often show a lot of fatty infiltration. In our image analysis pipeline, we performed a voxel-based analysis to obtain muscle microscopic fat infiltration and hydration values. Other statistical approaches can provide further information, with descriptors focusing on more extreme values (i.e., percentiles) or on texture and heterogeneity properties of the images, which have been proposed already to detect structural differences in the muscle, fat and bone marrow [13,65,66]. In addition, due to our sample size, it has not been possible to segment between sarcopenic and sarcopenic obesity subjects nor establish cut-off values or reference values for the different MRI key variables, requiring longitudinal and case-control studies to be carried out. Ranges of variation in BIA measurement observed could be due to the manufacturer of the measuring device. When considering the empirical data available for this device, there are limits between the BC-418 M and hydrostatic weighing (limits of agreement = 9% with hydrostatic weighing) [20]. Finally, although it provides multiple image contrasts and both morphological and functional images, MRI is not as accessible as other imaging modalities. In this context, recent studies have shown the promising role of ultrasound imaging as a tool to assess the amount and texture of fat in relation to muscle quality [67]. Further studies are needed to compare the outcome of both imaging modalities in order to establish optimal protocols that improve the diagnosis of sarcopenia.

## 5. Conclusions

In conclusion, this study provides a comprehensive analysis of skeletal muscle quality and quantity indicators, comparing clinical and biometrical parameters with MR-derived imaging biomarkers in a group of women with sarcopenia. Since the loss of muscle quality precedes the decrease in quantity, the knowledge of how these biomarkers behave can help to identify people at risk of developing sarcopenia at an early stage. This may allow taking preventive actions to decrease disability, morbidity, and mortality in older female sarcopenia patients. More studies are needed in order to establish cut off values or criterion-based reference values.

## Figures and Tables

**Table 1:** Imaging biomarkers obtained from MRI sequences.

MRI Sequence	Relevant Acquisition Parameters	Imaging Biomarker	Muscle Quality Indicator (Clinical Endpoint)
**T1-weighted turbo spin echo sequence (TSE-T1)**	Standard parameters	Total thigh muscle volume Total muscle volume/total fat volume Total muscle volume/total bone volume Macroscopic fatty infiltration	Muscle volume Muscle vs. fat ratio Muscle vs. bone ratio Macroscopic muscle fat infiltration
**Multiecho sequence with chemical shift**	Echo times: 0.88, 1.55, 2.22, 2.89, 3.56 and 4.23 ms	Microscopic proton density fat fraction (PDFF) [32] T2* relaxation time	Microscopic muscle fat infiltration Muscle hydration
**Diffusion-weighted sequence**	b-values: 0, 50, 100, 400 and 1200 s/mm^2^	Molecular diffusion of water: apparent diffusion coefficient (ADC) and diffusion coefficient (D) [33]	Muscle hydration

**Table 2:** Cineanthropometric, functional, respiratory, and image parameter characterization of the studied sample.

Parameter	Mean ± Standard Deviation (5th and 95th Percentiles)
**Cineanthropometric**	
Weight (kg)	62.7 ± 11.7 (47.0, 85.8)
Height (m)	1.50 ± 0.05 (1.42, 1.60)
BMI (kg/m^2^)	27.4 ± 4.7 (21.3, 36.8)
Thigh perimeter (cm)	47.4 ± 5.6 (42.1, 59.7)
Calf perimeter (cm)	32.5 ± 3.5 (27.8, 35.9)
Fat mass (kg)	24.0 ± 8.1 (12.7, 40.3)
Fat mass (%)	38.2 ± 7.5 (20.3, 48.4)
Muscle mass (total, kg)	36.2 ± 4.4 (29.8, 45.7)
Muscle mass (appendicular, kg)	15.4 ± 2.3 (12.9, 20.3)
Muscle mass (lower limbs, kg)	12.2 ± 1.3 (10.1, 15.8)
Skeletal muscle mass index (SMMI, kg/m^2^)	6.0 ± 0.8 (4.5, 7.1)
**Functional**	
Barthel index score (points)	95 ± 9 (65, 100)
SPPB (points)	7.0 ± 2.6 (3.4, 11.2)
MNA score (points)	14.0 ± 4.5 (12.0, 25.0)
IPAQ (Low/Moderate)	8/18
Gait speed (m/s)	0.72 ± 0.23 (0.27, 1.14)
Handgrip strength (kg)	18.0 ± 3.9 (11.8, 25.2)
Maximum isotonic knee extension (kg)	8.37 ± 3.1 (5.7, 13.3)
Maximum isotonic leg press (kg)	56.7 ± 24.3 (0, 93.4)
Maximum isometric knee extension (kg)	18.7 ± 6.4 (10.8, 28.7)
Mean isometric knee extension (kg)	16.5 ± 6.4 (9.6, 27.1)
**Respiratory**	
FVCa (L/s)	1.86 ± 0.57 (1.04, 2.71)
FVCp (%)	112.0 ± 24.5 (72.8, 150.8)
FEV1a (L/s)	1.46 ± 0.42 (0.82, 2.19)
FEV1p (%)	116.0 ± 26.6 (75.8, 172.3)
FEV2575a (L/s)	1.41 ± 0.69 (0.69, 2.81)
FEV2575p (%)	64.0 ± 29.5 (34.0, 125.5)
PEF (L/s)	3.7 ± 1.2 (1.5, 5.8)
MIP (cm H_2_O)	45.0 ± 20.3 (9.3, 74.5)
MEP (cm H_2_O)	77.0 ± 28.8 (35.3, 126.5)
**Muscle quality-related imaging biomarkers**	
Muscle hydration ADC (10^−3^ mm^2^/s)	1.05 ± 0.1 (0.81, 1.30)
Muscle hydration D (10^−3^ mm^2^/s)	1.01 ± 0.1 (0.65, 1.23)
Muscle hydration T2 (ms)	31.6 ± 4.1 (24.9, 37.1)
Muscle microscopic fat (PDFF, no units)	0.13 ± 0.03 (0.09, 0.20)
Macroscopic fatty infiltration (no units)	0.34 ± 0.10 (0.18, 0.56)
**Muscle quantity-related imaging biomarkers**	
Muscle/fat ratio (no units)	0.69 ± 0.30 (0.27, 1.23)
Muscle/bone ratio (no units)	13.0 ± 3.2 (8.1, 17.1)
Absolute muscle volume (L)	1.25 ± 0.4 (0.62, 2.05)

Abbreviations: BMI (body mass index), MNA (mini nutritional assessment), SPPB (short physical performance battery), FVCp (forced vital capacity), FEV1p (forced expiratory volume in 1 s), FEV2575p (forced expiratory volume at 25–75% of the pulmonary volume), PEF (peak expiratory flow), MIP (maximal inspiratory pressure), MEP (maximal expiratory pressure), ADC (apparent diffusion coefficient), D (diffusion coefficient), PDFF (proton density fat fraction), IPAQ (international physical activity questionnaire).

**Table 3:** Statistically significant correlations between imaging biomarkers and cineanthropometric parameters with |r| > 0.4.

	BMI (kg/m^2^)	Thigh Perimeter (cm)	Fat Mass (%)	Muscle Mass (Total, kg)	SMMI (kg/m^2^)
**Muscle hydration ADC (10^−3^ mm^2^/s)**	−0.71 ***	−0.32	−0.69 ***	−0.35	−0.23
**Muscle hydration D (10^−3^ mm^2^/s)**	−0.71 ***	−0.55 **	−0.72 ***	−0.62**	−0.51 *
Muscle hydration T2* (ms)	−0.62 **	−0.73 ***	−0.60 **	−0.25	−0.24
**Muscle microscopic fat (PDFF, no units)**	0.82 ***	0.29	0.74 ***	0.41*	0.37
**Macroscopic fatty infiltration (no units)**	0.67 ***	0.46 *	0.71 ***	0.24	0.10
**Muscle/fat ratio (no units)**	−0.61 **	−0.78 ***	−0.68 **	−0.35	−0.21
**Muscle/bone ratio (no units)**	−0.28	−0.14	−0.24	−0.12	0.02

Abbreviations: ADC (apparent diffusion coefficient), D (diffusion coefficient), T2 (transversal relaxation time), PDFF (proton density fat fraction), BMI (body mass index,), SMMI (skeletal muscle mass index). Significant * < 0.05; ** < 0.01; *** < 0.001.

**Table 4:** Statistically significant correlations between imaging biomarkers and functional parameters with |r| > 0.4.

	Handgrip (kg)	MeanISOMED (kg)	Max ISOMED (kg)	SPPB (Points)	MILP (kg)
**Muscle hydration ADC (10^−3^ mm^2^/s)**	−0.28	−0.36	−0.23	−0.28	−0.36
**Muscle hydration D (10^−3^ mm^2^/s)**	−0.28	−0.42	−0.45	0.02	−0.27
Muscle hydration T2* (ms)	− 0.27	−0.02	0.03	−0.23	−0.51 *
**Muscle microscopic fat (PDFF, no units)**	−0.51 *	−0.11	−0.21	−0.27	0.10
**Macroscopic fatty infiltration (no units)**	−0.10	−0.15	−0.20	−0.60 **	0.01
**Muscle/fat ratio (no units)**	−0.18	−0.57 *	−0.46 *	0.11	−0.18
**Muscle/bone ratio (no units)**	0.22	−0.42	−0.42 *	0.39 *	−0.28
**Absolute muscle volume (L)**	0.50 **	0.25	0.26	0.47 **	0.10

Abbreviations: ADC (apparent diffusion coefficient), D (diffusion coefficient), T2 (transversal relaxation time), PDFF (proton density fat fraction), Mean ISOMED (mean isometric knee extension), Max ISOMED (maximum isometric knee extension), SPPB (short physical performance battery), MILP (maximum isotonic leg press). Significant * < 0.05; ** < 0.01.

**Table 5:** Statistically significant correlations between imaging biomarkers and respiratory parameters with |r| > 0.4.

	FVCa (L/s)	FEV1a (L/s)	FEV2575a (L/s)	PEF (L/s)	MIP (H_2_Ocm)	MEP (H_2_Ocm)
**Muscle hydration ADC (10^−3^ mm^2^/s)**	−0.44 *	−0.56 **	−0.61 **	−0.26	−0.54 **	−0.30
**Muscle hydration D (10^−3^ mm^2^/s)**	−0.26	−0.33	−0.55 **	−0.18	−0.10	−0.10
Muscle hydration T2* (ms)	−0.36	−0.30	−0.54 *	−0.10	0.17	−0.37 *
**Muscle microscopic fat (PDFF, no units)**	−0.21	−0.13	−0.20	0.03	0.21	0.08
**Macroscopic fatty infiltration (no units)**	−0.65 ***	−0.48 *	−0.32	−0.51 **	−0.37	−0.25
**Muscle/fat ratio (no units)**	−0.28	−0.29	−0.23	−0.15	−0.35	−0.24
**Muscle/bone ratio (no units)**	0.47 *	0.38	0.29	0.18	−0.28	−0.01
**Absolute muscle volume (L)**	0.53 **	0.09	0.18	0.47 *	0.36	0.32 *

Abbreviations: ADC (apparent diffusion coefficient), D (diffusion coefficient), T2 (transversal relaxation time), PDFF (proton density fat fraction, FVCa (forced vital capacity), FEV1a (forced expiratory volume in 1 s), FEV2575a (forced expiratory volume at 25–75% of the pulmonary volume), PEF (peak expiratory flow, L/s), MIP (maximal inspiratory pressure), MEP (maximal expiratory pressure). Significant * < 0.05; ** < 0.01; *** < 0.001.
